# Mental Health Help-Seeking Among Young Internal Migrants in China: Shame as a Double-Edged Sword

**DOI:** 10.1007/s11126-025-10127-9

**Published:** 2025-04-15

**Authors:** Xuhong Li, Kin-Kit Li

**Affiliations:** Department of Social and Behavioural Sciences, City University of Hong Kong, Tat Chee Avenue, Kowloon, Hong Kong SAR China

**Keywords:** Migrants, Shame, Help-seeking, Stress, Mental health

## Abstract

Young migrants in mainland China are vulnerable to mental health issues and are reluctant to seek help from professional sources. Shame is considered an important culture-specific emotion affecting professional help-seeking. The current study aimed to investigate the moderating role of shame in the association between psychological stress and help-seeking in young internal migrants in China. 415 internal migrants (mean age = 29.20; SD = 4.81) were recruited to participate in the survey study with oversampling of those who sought help before. Multiple linear regression and Zero-inflated Poisson regression models were used to examine the moderating effects of shame on the stress-intention and stress-behavior relations, respectively. Among the 65.0% of participants with high psychological stress, 62.6% of them never sought professional help for their mental health issues. The results demonstrate that shame significantly reduced the positive association between psychological stress and professional help-seeking behavior. Conversely, shame also played a protective role, mitigating the negative association between psychological stress and professional help-seeking intention. The findings underline the importance of addressing shame-related issue prior to therapy and developing promotion strategies to improve young migrants’ mental health.

**Supplementary Information:**

The online version contains supplementary material available at 10.1007/s11126-025-10127-9.

## Introduction

With rapid urbanization globally, a significant proportion of residents migrate from rural to urban areas in search of employment opportunities. This phenomenon, known as internal migration, is particularly prevalent in developing countries in Asia, South America, and Africa [1]. Existing international research has consistently shown that internal migration can result in negative health outcomes and a reduced quality of life [2, 3]. However, many studies on health-related issues have been descriptive and focused primarily on international migrants, paying less attention to the impacts of internal migration and its underlying mechanisms [4, 5].

Over the past four decades, China has experienced rapid economic growth and urbanization, resulting in significant internal population flows from rural villages to urban areas [6]. Unlike their counterparts in other countries, internal migrants in China are regarded as temporary residents or “guest workers” because they are not entitled to healthcare and social service in host cities due to the household registration system (hukou system), which plays an institutional role in social resource allocation and population management [6, 7]. Empirical evidence shows that internal migrants face several difficulties in the cities where they seek a higher quality of life, such as demanding working environments, limited access to social welfare benefits, and discrimination [7–9]. Many of them live in overcrowded housing and work in intensive but low-paying occupations, such as construction, service industries, and factory job, which subsequently increases their risk of poor physical and mental health outcomes [8]. According to [10], nearly half of the migrants are young (under 40 years old), which is the peak age for the onset of mental health problems [11, 12].

Although young migrants are vulnerable to mental illness, the understanding of mental health help-seeking among this disadvantaged group remains limited. Shame has been identified as an important factor influencing mental health help-seeking intentions and behaviors [13, 14]. This study examined whether and how shame moderates the relation between stress and help-seeking intentions, and the relation between stress and help-seeking behaviors.

The theory of psychological disharmony posits that higher work-related stress and less acculturation catalyze the development of mental health problems among migrants [15, 16]. Compared to older migrants, young migrants are often separated from their previous social networks for the first time and face difficulties in urban integration [17]. Additionally, demanding work, poor social support, and limited access to cultural activities may contribute to self-sacrifice and low awareness of self-care, leading to psychological stress [18]. A recent study found that at least 30% of young migrants had common mental health problems, a rate higher than the general population’s 17.5% [19]. Moreover, the prevalence of lifetime suicidal ideation among Chinese migrants (12.8%) was much higher than that of the general public (3.9%) [20]. However, young migrants were less likely to seek help for mental health problems compared with non-migrants [17].

Many factors contributing to the reluctance to seek professional help have been evident in previous studies. These include being male, having a poor social network, limited understanding of mental illness, stigma, and a perception of hopelessness [17, 21, 22]. While much of the research to date has primarily focused on biological and social factors affecting professional help-seeking for mental health problems, there have been limited empirical investigations into the role of psychological risk factors. Achieving a deeper understanding of factors that contribute to professional help-seeking in young migrants would guide mental health professionals in designing help-seeking promotion campaigns.

According to Andersen Behavioral Model (ABM) [23], factors influencing help-seeking behavior can be categorized as 1) predisposing factors – varying inclination to seek help, 2) enabling factors – capacity to obtain resources, and 3) need factors – the necessity for seeking help [24]. Specifically, perceived need factors, represented by psychological stress, appear to be the most important determinants of seeking treatment [25, 26]. Notably, previous research has yielded contradictory results. Some studies have shown a positive association between psychological stress and professional help-seeking [27, 28]. However, these findings contradict other studies [29–31], which indicate that individuals with psychological issues might refuse to seek help from professionals, particularly among younger adults.

It is plausible that some predisposing personal characteristics might interact with psychological stress to influence the help-seeking process [32]. In other words, certain predisposing personal characteristics may moderate the association between psychological distress and help-seeking. Previous literature has found that shame has a robust impact on mental health recovery [13, 33–35]; it represents a significant emotional construct associated with mental health [36, 37]. Shame is not solely a Chinese or Asian phenomenon; it is also widely reported in many other cultures [38]. However, cross-cultural studies on shame have highlighted the differences in its manifestation [39, 40]. In Western culture, shame is more likely to imply the feelings of regret and compunction, namely guilt incurred by individuals’ own negative self-evaluation [41–43]. In contrast, shame in Eastern cultures often involves the perception of how others view the individual [44]. In East Asian societies (e.g., China and Japan), where collectivism is highly valued, individuals’ shameful behavior is conceived as a group, family, or community issue, embedded in the moral and reputational aspects [40, 45]. In such societies, individuals usually fear that their inadequacies or abnormalities might lead to being rejected and excluded by their groups, given that social order and conformity are strongly emphasized [39].

Shame, as a painful feeling, can maintain or exacerbate psychological symptoms by reinforcing a sense of deficiency, powerlessness or worthlessness [42, 46, 47]. According to the self-regulation theory perspective, shame may serve to activate, maintain, or inhibit individuals’ intention and behavior in response to internal cues and environmental stimuli [48]. Specifically, shame experiences play a role in self-regulatory processes that discourage individuals from engaging in behaviors which violate social norms or motivate them to move away from the shame-inducing situations in collectivist cultures [49, 50].

A qualitative study conducted among Asian women reported that seeking help outside the family is considered damaging to family honor and brings shame [44]. Moreover, individuals who have a family member with a mental disorder might suffer from shame pertinent to stigma, consequently leading to avoidance of disclosing mental health problems [51]. It appears that shame might reduce the positive relationship between psychological distress and professional help-seeking behavior. However, the findings regarding help-seeking intention are inconsistent. Some empirical findings suggested that shame has an inhibiting effect between psychological stress and help-seeking intention by promoting avoidant coping strategies [13, 52]. Other studies have found that individuals might display an intention to seek help if they experience a sense of shame, driven by the motivating power of shame in personal change [53, 54]. In summary, it is plausible that shame plays a moderating role in the association between mental health and help-seeking.

In light of the increased attention to cultural mechanisms of mental health, it is urgent to deepen our understanding of shame in promoting professional help-seeking. This study aims to examine the role of shame in the association between psychological distress and professional help-seeking in young migrants in southern China.

Notably, previous studies highlighted the necessity of distinguishing between intention and actual behavior in professional help-seeking, even though a strong link between help-seeking behavior and intention has been reported [55, 56]. For this reason, this study examines both professional help-seeking behavior and intention. It is hypothesized that shame amplifies the stress-intention relation but buffers the stress-behavior relation.

Andersen Behavioral Model (ABM) [23], a comprehensive and widely used framework, was employed to guide the analysis of the current study. It posits that factors affecting individuals’ decisions for health-related behaviors can be categorized into predisposing factors, enabling factors and need factors. In this study, predisposing factors included age, gender, marital status and educational level. Additionally, the length of living in the current city and hukou status were also considered predisposing factors, reflecting the unique characteristics of the migrant population. Enabling factors encompassed income, health insurance coverage, and social network. Apart from psychological stress, need factors also included general health conditions.

## Methods

### Sample

A cross-sectional online survey was conducted from March to April 2022, in Guangdong province, China. We purposively recruited participants from multiple sources, including a factory, a community centre, and two healthcare centres. While the convenient sample from the factory should be the most representative of the young migrant population, individuals seeking professional help are relatively rare. Therefore, we also recruited participants from community centres and healthcare centres to increase the number of participants who sought professional help. This oversampling technique increased the statistical power for predicting a rare outcome and improved the representation of this small segment of the population.

The survey link of the study was distributed with the assistance of a community centre, two healthcare centres, and a factory through various information dissemination platforms, such as Wechat groups, flyers, and posters. In the factory, department managers were contacted to facilitate the distribution of the survey link. In the community and healthcare centres, some doctors and social workers helped disseminate the survey information to potential participants. Additionally, participants at each site were encouraged to share the survey link with their peers who met the inclusion criteria.

To verify participants’ eligibility, individuals were required to report their place of birth and the city where they were working. Those who indicated that they were local residents were excluded. The inclusion criteria of participants included: 1) being migrants in the current cities at least one year, and 2) being aged 18–40 years. All participants had to provide their consent before starting the survey, which took approximately 15–20 min to complete on average. The current study gained ethical approval from the institutional review board of City University of Hong Kong (Approval Code: RPG-2022–48).

### Measurement

#### Dependent Variable-Professional Help-Seeking

Actual professional help-seeking behavior was measured by the number of times participants sought professional help from mental health professionals, such as counselors and psychiatrists, in the past 12 months. Additionally, the Mental Help Seeking Intention Scale (MHSIS) [57] was used to measure participants’ intention to seek help from mental health professionals. This scale consists of 3 items rated on a 7-point Likert scale, ranging from 1 (definitely false) to 7 (definitely true). An example item is, “If I had a mental health concern, I would intend to seek help from a mental health professional.” A higher score indicates a stronger intention to seek professional help. A reliability test revealed a high level of internal consistency (Cronbach’s alpha = 0.84).

#### Independent Variable-Psychological Stress

Psychological stress was measured using the Kessler Psychological Distress Scale (K6), a brief and reliable tool for screening participants’ symptoms [58]. Participants rated each item on a 5-point Likert-type scale, ranging from 1 (none of the time) to 5 (all of the time). An example question is, “How often did you feel hopeless?” A higher score indicates greater psychological stress. The Chinese version of the K6 demonstrated satisfactory validity and reliability [59]. In the current study, it achieved excellent internal consistency (Cronbach’s alpha = 0.93).

#### Shame

Shame was evaluated using the Shame Proneness Scale, which was modified from the Personal Feelings Questionnaire 2 [38, 60, 61]. This scale consists of 30 items that assess participants’ proneness to shame. The items are categorized according to three types of social partners: family members, friends, and strangers, with the same items used for each partner type. Each item is rated on a 5-point scale, ranging from 1 (far less than usual) to 5 (far more than usual). An example question is, “How often do you feel embarrassed?”. The total score is obtained by summing all items, with a higher score indicating a stronger proneness to shame. The scale was translated to Chinese using the forward–backward approach [62]. First, the English version of the scale was translated to Chinese by two social science researchers independently. Any conflict and discrepancy were discussed and resolved to produce a final translated version. Second, it was back translated to English by a researcher who was proficient in both English and Chinese with psychology background, but was not familiar with the original scale. Any discrepancies were addressed through consensus. Finally, a pilot test was conducted among 10 young migrants to ensure clarity. In the current study, the scores achieved a Cronbach’s alpha of 0.98.

#### Covariates

As mentioned above, the predisposing factors consisted of age, gender, marital status (married/not married), educational level (high school or lower, vocational school, and tertiary education), the length of residence in the current city (years) and hukou status (whether the individual has citizenship in the working city). The enabling factors consisted of household income, health insurance coverage (yes/no) and social network. Social network was measured using the Lubben Social Network Scale (LSNS-6; [63]. Each item was scored on a 5-point Likert scale, with higher total scores indicating a greater social network. The additional need factor, general health condition, was evaluated by asking participants to self-rate their health with the question, “Would you describe your health in general?” on a 5-point scale ranging from 1 (poor) to 5 (excellent). A higher score indicates better perceived health.

### Analytic Strategy

All statistical analyses were computed using SPSS version 26 and Mplus version 8. Descriptive analysis was employed to describe the characteristics of young migrants. Correlation analyses were conducted to assess the relationships between psychological stress, shame, and professional help-seeking.

To examine whether shame moderates the relationship between psychological stress and professional help-seeking intention, a moderated multiple regression analysis was conducted. Additionally, Zero-inflated Poisson (ZIP) regression model was employed to explore the moderating effects of shame on the relationship between psychological stress and professional help-seeking behavior. This approach was used because the number of times participants sought professional help is a count variable, and there is a notable excess of zero values, indicating a significant number of participants without professional help-seeking experience in the past year [64]. The ZIP regression model effectively accounts for data with excess zero values by implementing two interrelated regression models.

Specifically, psychological stress and shame were entered into the regression models first, followed by the interaction term between psychological stress and shame. The covariates mentioned above were included in the models. To illustrate the interaction between psychological stress and shame in relation to professional help-seeking, the regressions of psychological stress on professional help-seeking were plotted at low and high levels of shame (Mean − 1SD and Mean + 1SD).

## Results

### Sample Characteristics

A total of 415 participants completed the questionnaire survey, of which 56.1% were married. The sample included 45.1% female participants, with a mean age of 29.2 years (SD = 4.81). A small number reported relatively low monthly personal incomes (< CNY 4,000). Surprisingly, about half of them had received tertiary education. More than three-quarters did not possess citizenship (hukou) in the city where they were working and living. The average length of residence in the current city was 4.93 years (SD = 3.41). Moreover, among the 65% of participants experiencing high psychological stress (mean ≥ 2.17), 62.6% reported never having sought help from a mental health professional in the past 12 months. Table 1 presents the sociodemographic characteristics and professional help-seeking behaviors of the participants.

**Table 1:** Sociodemographic characteristics of participants

	*N* (Mean)	% (SD)
*Predisposing factors*		
Age	(29.2)	(4.81)
Gender		
Men	228	54.9
Women	187	45.1
Marital status		
No married	182	43.9
Married	233	56.1
Education level		
≤ High school	97	23.4
Vocational school	102	24.6
Tertiary education	216	52
Hukou status		
No	323	77.8
Yes	92	22.2
Length of residence in the current city	(4.93)	(3.41)
*Enabling factors*		
Monthly Income		
0–3,999 CNY	11	2.7
4,000–7,999 CNY	50	12
8,000–11,999 CNY	75	18.1
12,000–15,999 CNY	113	27.2
16,000–19,999 CNY	61	14.7
≥ 2,0000 CNY	105	25.3
Insurance coverage		
Yes	386	93
No	29	7
*Need factors*		
General health condition		
Excellent	151	36.4
Very good	114	27.5
Good	68	16.4
Fair	68	16.4
Poor	14	3.4
Professional help-seeking behavior		
Yes	157	37.8
No	258	62.2

### Correlations Between Main Variables

As shown in Table 2, professional help-seeking intention was significantly associated with shame (*r* = −0.31, *p* < 0.001) and psychological stress (*r* = −0.28, *p* < 0.001), respectively. Furthermore, shame was strongly associated with psychological stress (*r* = 0.73, *p* < 0.001). A significant relationship between intention and actual behavior was also observed (*r* = 0.23, *p* < 0.001).

**Table 2:** Mean, standard deviations (SD) and correlations

	Mean	SD	1	2	3	4
1.Psychological stress	2.65	0.98	1			
2.Shame	2.39	0.9	.728**	1		
3.Professional help-seeking intention	5.66	1.00	-.279**	-.309**	1	
4.Professional help-seeking behavior	1.03	1.86	.038	-.055	.237**	1

Note: **p ＜ 0.001

### Moderating Effect Analyses

In terms of actual professional help-seeking behavior, the results were generated simultaneously using two separate models (count and logistic model). Table 3 presents the results from zero-inflated Poisson regression analyses. The results of the count model showed that being female (b = −0.27, *p* = 0.03) and general health (b = −0.19, *p* < 0.001) were negatively associated with professional help-seeking behavior, while length of living in the current city (b = 0.05, *p* = 0.02) was positively associated with professional help-seeking behavior. Additionally, the results of the logistic model showed that being female (b = −0.92, *p* = 0.018) and income (b = −1.26, *p* < 0.001) were negatively associated with professional help-seeking behavior.

**Table 3:** Zero-inflated Poisson regression for professional help-seeking behavior

Count model	b	SE	*p*	IRR
Psychological stress	0.89	0.26	0.001	2.44
Shame	0.88	0.35	0.012	2.42
Psychological stress × Shame	−0.28	0.11	0.007	0.75
*Predisposing factors*				
Age	0.01	0.02	0.567	1.01
Women	−0.27	0.12	0.030	0.77
Married	0.16	0.14	0.276	1.17
Vocational education	−0.34	0.16	0.041	0.72
Tertiary education	−0.46	0.15	0.003	0.63
Hukou status	−0.12	0.20	0.555	0.89
Length of residence in the current city	0.05	0.02	0.002	1.05
*Enabling factors*				
Income	−0.03	0.07	0.617	0.97
Insurance coverage	0.62	0.24	0.011	1.86
Social network	−0.04	0.01	0.547	0.96
*Need factor*				
General health condition	−0.19	0.05	0.000	0.83
Logistic (Zero-inflated) model	b	SE	*p*	OR
Psychological stress	0.93	0.79	0.240	1.17
Shame	1.81	0.95	0.057	1.20
Psychological stress × Shame	−0.42	0.29	0.153	0.99
*Predisposing factors*				
Age	0.14	0.05	0.003	1.15
Women	−0.92	0.39	0.018	0.40
Married	−0.72	0.39	0.061	0.49
Vocational education	−0.74	0.46	0.110	0.48
Tertiary education	−0.07	0.42	0.871	0.93
Hukou status	0.10	0.44	0.827	1.10
Length of residence in the current city	−0.09	0.05	0.041	0.91
*Enabling factors*				
Income	−1.26	0.35	0.000	0.28
Insurance coverage	1.21	0.88	0.171	3.35
Social network	0.10	0.20	0.605	1.11
*Need factor*				
General health condition	0.49	0.15	0.001	1.62

**Fig. 1:**
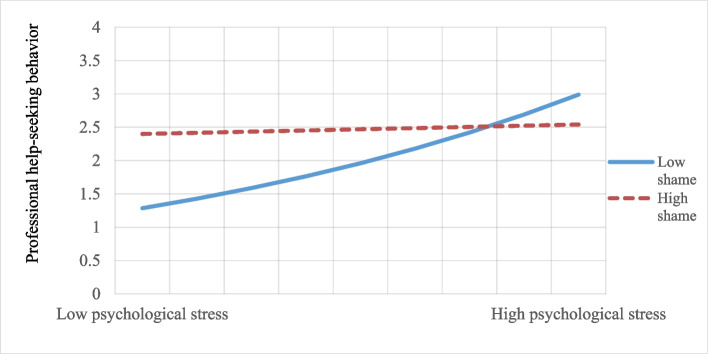
The moderating effect of shame in the relationship between psychological stress and professional help-seeking behavior

Furthermore, psychological stress (b = 0.89, *p* = 0.001) and shame (b = 0.88, *p* = 0.012) were positively associated with professional help-seeking behavior, while the interaction of psychological stress with shame was significantly negative (b = −0.28, *p* = 0.007) in the count model. This indicates that shame moderated and buffered the association between psychological stress and actual help-seeking behavior. As shown in Fig. 1, at lower levels of psychological stress, help-seeking behaviors were higher in the high shame condition, while at higher levels of psychological stress, help-seeking behaviors were higher in the low shame condition. However, the interaction in the logistic model did not show a significant effect.

**Fig. 2:**
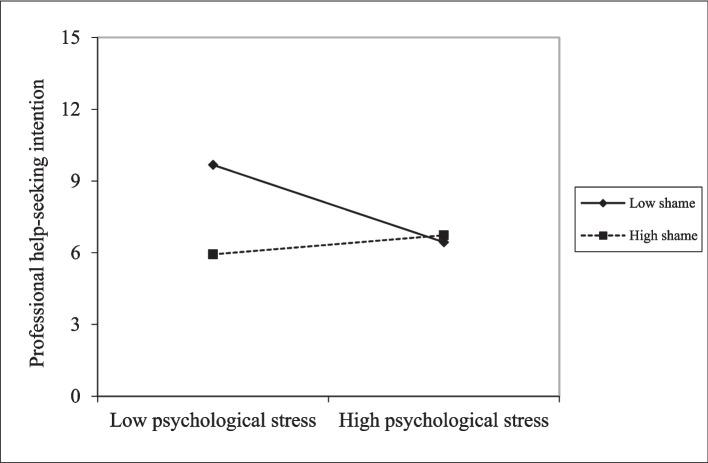
The moderating effect of shame in the relationship between psychological stress and professional help-seeking intention

As shown in Table 4, previous help-seeking behavior (b = 0.21, *p* = 0.001), social network (b = 0.26, *p* < 0.001) and general health (b = −0.15, *p* = 0.002) were significantly linked to professional help-seeking intention. Figure 2 illustrates that lower levels of psychological stress were associated with higher help-seeking intention in the low shame condition. Furthermore, psychological stress (b = −0.62, *p* < 0.001) and shame (b = −0.96, *p* < 0.001) were negatively associated with professional help-seeking intention. However, a significant positive interaction effect of psychological stress × shame (b = 1.14, *p* < 0.001) was observed, indicating that shame moderated and amplified the relationship between psychological stress and professional help-seeking intention. This finding was consistent with sensitivity analyses, which also revealed a positive interaction effect of psychological stress × shame (b = 1.44, *p* < 0.001) (Supplementary Table 1 and Supplementary Fig. 1), even after excluding participants who had sought help before.

**Table 4:** Regression models for professional help-seeking intention

	b	SE	*p*
Psychological stress	−0.62	0.13	0.000
Shame	−0.96	0.15	0.000
Psychological stress × Shame	1.14	0.23	0.000
*Predisposing factors*			
Age	0.04	0.05	0.428
Women	0.05	0.04	0.187
Married	0.05	0.05	0.305
Vocational education	0.11	0.05	0.031
Tertiary education	0.03	0.06	0.551
Hukou status	−0.04	0.05	0.391
Length of residence in the current city	0.06	0.04	0.144
Professional help-seeking behavior	0.21	0.06	0.001
*Enabling factors*			
Income	−0.03	0.05	0.589
Insurance coverage	0.03	0.05	0.540
Social network	0.26	0.06	0.000
*Need factor*			
General health condition	−0.15	0.05	0.002

## Discussion

To the best of our knowledge, this was the first study investigating the moderating role of shame in professional help-seeking among young migrants in mainland China. It contributes to broadening the understanding of professional help-seeking and shame in Chinese societies. The results demonstrate that the majority of participants (64.4%) experienced high levels of psychological stress [65]. However, the prevalence of professional help-seeking behaviors among participants was relatively low, which is consistent with the previous findings [17, 66]. The robust associations between shame and professional help-seeking intention as well as behavior broadly support the earlier observations that shame plays an important role in professional help-seeking to varying degrees [13, 35, 36].

Notably, the length of residence in the current city was significantly associated with professional help-seeking behavior among those who had sought help. This may be attributed to the idea that a longer stay is linked to a better awareness of available services, thereby facilitating more consistent help-seeking [67]. In addition, Millán-Franco and the colleagues [68] demonstrated that migration often led to social exclusion. Ensuring appropriate intervention for migrants, promoting their social inclusion, and improving their awareness of services should be key objectives for mental health services.

According to the ABM, need factors act as the most robust predictors of health-related behaviors [69]. In the current study, the need factors, conceptualized as psychological stress and general health condition, play important roles in both professional help-seeking intention and behavior. Psychological stress was negatively associated with professional help-seeking intention, which is consistent with a previous study [55] demonstrating that psychological issue, such as a sense of hopelessness, may decrease positive coping tendencies. However, greater psychological stress increased the odds of professional help-seeking behavior, as young migrants might perceive stress as a risk factor that hinders their daily functioning [70]. In mainland China, migrant workers’ financial support is often the primary source of income for their children and parents [71]. Consequently, they may seek professional help for psychological issue driven by the need to continue working, especially considering the long-term detrimental effects of psychological symptoms on their work capacity.

The results showed that shame moderated the association between psychological stress and actual help-seeking behavior. Notably, among participants who have sought professional help, shame acted as a buffering agent between psychological stress and professional help-seeking behavior. Empirical findings suggest that shame contributes to self-sigma regarding mental health issue, which arises when individuals with mental health problems internalize public stigma [13]. In Chinese societies, where collectivism is highly emphasized, seeking external help for mental health problems is often viewed as a source of dishonor and embarrassment for individuals and their families [14, 72].

This aligns with previous findings that shame is a universally painful and dysregulated emotion that can lead to withdrawal behavior when individuals feel they cannot meet internal or external expectations [73]. Consequently, the young migrants who are experiencing shame may consistently refuse to seek professional help. Another possible interpretation is that young migrants may prefer to seek help from informal sources, such as family members and friends, instead of accessing formal sources [17, 74]. Additionally, Chinese young migrants were generally excluded from the social welfare system in their host cities [8, 9], resulting in greater concern about disclosing their personal information and fear of facing stigma while seeking professional help for sensitive health issues [17, 72]. The observation that help-seeking behaviors were higher in the low shame condition at higher levels of psychological stress could be attributed to individuals perceiving their psychological issue as less shameful when they receive positive, useful and accurate information through increased professional help-seeking behavior [75].

Regarding professional help-seeking intention, the moderation analyses demonstrated that psychological stress elicited an avoidance intention, whereas shame served a protective role, mitigating such association. This may be attributed to the fact that individuals who feel and express more shame are more likely to desire self-change; in other words, shame acts as a significant motivator for individuals to alter or control their situations [76].

This finding extends the previous research indicating that experience of shame motivates younger individuals to restore their damaged image, as psychological issues are viewed as moral bankruptcy and associated with the loss of a respectable image in Chinese societies. This perception subsequently activates the intention to address psychological issues [77–80]. Another possible explanation is that shame may arise from interruptions in employment status and failures to support their families due to psychological issues. Therefore, migrants might have a strong intention to address the psychological concerns [20, 81].

It is worth noting that even among the low shame groups, there was no significant decrease in professional help-seeking intention when they perceived lower levels of psychological stress. This suggests that they may consider help-seeking acceptable and beneficial while distinguishing themselves from individuals facing severe psychological issues [82]. Other possible interpretations include the possibility that some participants rated the option due to social desirability or exhibited overconfidence in this area. Future studies could benefits from using in-depth interviews (e.g., open-ended question) to investigate this further [83].

The inconsistent outcome between intention and actual behavior in the current study support the notion that shame may function as a paradoxical double-edged sword due to its complexity. As a global attribution, shame can evoke self-change motivation aimed at avoiding pain and restoring the self, thereby increasing the intention to seek help. Conversely, as an emotional proxy for stigma, it may induce avoidance-oriented behavioral responses [76, 77, 84].

In line with previous research [55], the findings support a significant link between professional help-seeking behavior and intention. Although psychological stress negatively influenced intention, a positive link between psychological stress and behavior was observed. This result might be partly attributed to the fact that severe psychological symptoms, such as suicidal thoughts, anhedonia, and sleeping problems, can serve as triggers to seek help [21]. Additionally, it is likely that some individuals may perceive their symptoms caused by the psychological stress as a sign of physical issues. Therefore, they would not hesitate to access primary health care, subsequently being referred to mental health professionals [85, 86]. Furthermore, the findings show that individuals with positive treatment experiences usually show more favorable intentions toward professional help-seeking. Evidence suggests that establishing trusted and supportive relationships between young people and mental health professionals is crucial in promoting their further professional help-seeking [87].

Some limitations of the current study should be noted. First, the cross-sectional design prevents the inference of causal relationships between professional help-seeking and its influencing factors. Second, it is impossible to avoid the recall bias in actual help-seeking behavior in the past 12 months, as it can be difficult for participants to accurately recall the past experiences, which might lead to underestimate or overestimate the moderating effect of shame. In addition, social desirability bias could influence participants to provide socially desirable answers rather than accurate responses, potentially resulting in the underreporting of stress. Third, the sample investigated was relatively limited and not representative of the broader population. Despite these limitations, a multi-source data collection with the oversampling of participants who sought professional help contribute to a better representation of this small subset of the population.

## Conclusion

In conclusion, internal migration among young individuals in developing countries is common and largely driven by economic factors. Many young migrants were exposed to vulnerable socio-economic circumstances as well as challenges in maintaining their physical and mental health. The findings accentuate the momentousness of considering a common but powerful emotional reaction when promoting migrants’ health and quality of life worldwide.

The prevalence of mental health problems and lower rate of professional help-seeking among young migrant workers highlight the need for effective promotional strategies and increased access to public mental health services. This study contributes to the literature by emphasizing the key role of shame as an underlying emotional mechanism influencing professional help-seeking among young migrants. It underscores the moderating effect of shame in the association between psychological stress and professional help-seeking.

Mental health professionals should be aware of the potential emotional issues faced by young migrant workers, addressing their feelings of shame prior to treatments, as such feelings may hinder the effectiveness of treatment [72]. Given that coping with stigma-related stress associated with shame can help reduce young people’ risk of mental illness, greater efforts are needed to promote anti-stigma programmes [52]. Furthermore, future research utilizing qualitative methods to gain a deeper and more comprehensive understanding of this area is strongly recommended.

## Supplementary Information

Below is the link to the electronic supplementary material.

Supplementary file1 (DOCX 27 KB)

## Data Availability

The data that support the findings of this study is available from the first author upon reasonable request.

## References

[1] Raghuraman BS, Chaturvedi S. Internal migration. In: Bhugra D, Ayonrinde O, Tolentino EJ, Valsraj K, Ventriglio A, Bhugra D, editors. Oxford Textbook of Migrant Psychiatry. Oxford University Press; 2021. p. 0.

[2] Kratz F. On the way from misery to happiness? A longitudinal perspective on economic migration and well-being. Migr Stud. 2020;8:307–55.

[3] Stawarz N, Arránz Becker O, Rüger H. Work-related internal migration and changes in mental and physical health: A longitudinal study using German data. Health Place. 2022;75:102806. 35533591 10.1016/j.healthplace.2022.102806

[4] Li J, Rose N. Urban social exclusion and mental health of China’s rural-urban migrants – A review and call for research. Health Place. 2017;48:20–30. 28892746 10.1016/j.healthplace.2017.08.009

[5] Liao L, Du M, Chen Z. Environmental pollution and socioeconomic health inequality: Evidence from China. Sustain Cities Soc. 2023;95:104579.

[6] Zhuang XY, Wong DFK. Differential impacts of social support on mental health: A comparison study of Chinese rural-to-urban migrant adolescents and their urban counterparts in Beijing. China Int J Soc Psychiatry. 2017;63:48–56. 27856949 10.1177/0020764016678015

[7] Meng X, Xue S. Social networks and mental health outcomes: Chinese rural–urban migrant experience. J Popul Econ. 2020;33:155–95.

[8] Wong DFK, Chang Y-L. Mental health of Chinese migrant workers in factories in Shenzhen, China: Effects of migration stress and social competence. Soc Work Ment Health. 2010;8:305–18.

[9] Wu Q. Effects of social capital in multiple contexts on the psychosocial adjustment of Chinese migrant children. Youth Soc. 2017;49:150–79.

[10] National Bureau of Statistics of China. Statistical Communiqué of the People’s Republic of China on the 2021 national economic and social development. Beijing, China: National Bureau of Statistics of China; 2022.

[11] Kessler RC, Demler O, Frank RG, Olfson M, Pincus HA, Walters EE, et al. Prevalence and treatment of mental disorders, 1990 to 2003. N Engl J Med. 2005;352:2515–23. 15958807 10.1056/NEJMsa043266 PMC2847367

[12] Lynch L, Long M, Moorhead A. Young men, help-seeking, and mental health services: Exploring barriers and solutions. Am J Mens Health. 2018;12:138–49. 27365212 10.1177/1557988315619469 PMC5734535

[13] Rüsch N, Müller M, Ajdacic-Gross V, Rodgers S, Corrigan PW, Rössler W. Shame, perceived knowledge and satisfaction associated with mental health as predictors of attitude patterns towards help-seeking. Epidemiol Psychiatr Sci. 2014;23:177–87. 23866069 10.1017/S204579601300036X PMC6998175

[14] Sangar M, Howe J. How discourses of sharam (shame) and mental health influence the help-seeking behaviours of British born girls of South Asian heritage. Educ Psychol Pract. 2021;37:343–61.

[15] Boski P. A psychology of economic migration. J Cross-Cult Psychol. 2013;44:1067–93.

[16] Castañeda H, Holmes SM, Madrigal DS, Young M-ED, Beyeler N, Quesada J. Immigration as a social determinant of health. Annu Rev Public Health. 2015;36:375–92. 25494053 10.1146/annurev-publhealth-032013-182419

[17] Yu C, Lou C, Cheng Y, Cui Y, Lian Q, Wang Z, et al. Young internal migrants’ major health issues and health seeking barriers in Shanghai, China: a qualitative study. BMC Public Health. 2019;19:336. 30902080 10.1186/s12889-019-6661-0 PMC6431074

[18] Idemudia E, Boehnke K. Psychosocial Experiences of African Migrants in Six European Countries: A Mixed Method Study. Cham: Springer International Publishing; 2020.

[19] Zhong BL, Liu TB, Chan SSM, Jin D, Hu CY, Dai J, et al. Common mental health problems in rural-to-urban migrant workers in Shenzhen, China: prevalence and risk factors. Epidemiol Psychiatr Sci. 2018;27:256–65. 28067189 10.1017/S2045796016001141 PMC6998856

[20] Zhang K, Xu C, Zhang Y, Wang R, Yu X, Hu T, et al. The mental health and syndemic effect on suicidal ideation among migrant workers in China: A cross-sectional study. Int J Environ Res Public Health. 2021;18:11363. 34769881 10.3390/ijerph182111363 PMC8583422

[21] Eigenhuis E, Waumans RC, Muntingh ADT, Westerman MJ, Van Meijel M, Batelaan NM, et al. Facilitating factors and barriers in help-seeking behaviour in adolescents and young adults with depressive symptoms: A qualitative study. PLoS ONE. 2021;16:e0247516. 33684154 10.1371/journal.pone.0247516 PMC7939362

[22] Koydemir-Özden S, Erel Ö. Psychological help-seeking: role of socio-demographic variables, previous help-seeking experience and presence of a problem. Procedia - Soc Behav Sci. 2010;5:688–93.

[23] Andersen RM. Revisiting the behavioral model and access to medical care: Does it matter? J Health Soc Behav. 1995;36:1. 7738325

[24] Andersen R, Newman JF. Societal and individual determinants of medical care utilization in the United States. Milbank Q. 2005;83. 10.1111/j.1468-0009.2005.00428.x 4198894

[25] Andrea M, Dias Ó, Andrea M, Figueira ML. Functional voice disorders: The importance of the psychologist in clinical voice assessment. J Voice. 2017;31:507.e13-507.e22. 27876300 10.1016/j.jvoice.2016.10.013

[26] Haarasilta L, Marttunen M, Kaprio J, Aro H. Major depressive episode and health care use among adolescents and young adults. Soc Psychiatry Psychiatr Epidemiol. 2003;38:366–72. 12861442 10.1007/s00127-003-0644-1

[27] Aldalaykeh M, Al-Hammouri MM, Rababah J. Predictors of mental health services help-seeking behavior among university students. Cogent Psychol. 2019;6:1660520.

[28] Yamaguchi S, Wu S-I, Biswas M, Yate M, Aoki Y, Barley EA, et al. Effects of short-term interventions to reduce mental health-related stigma in university or college students: A systematic review. J Nerv Ment Dis. 2013;201:490–503. 23719324 10.1097/NMD.0b013e31829480df

[29] Babitsch B, Gohl D, Von Lengerke T. Re-revisiting Andersen’s behavioral model of health services use: A systematic review of studies from 1998–2011. GMS Psycho-Soc-Med 9Doc11 ISSN 1860–5214. 2012. 10.3205/PSM000089. 10.3205/psm000089 PMC3488807 23133505

[30] Rüsch N, Lieb K, Göttler I, Hermann C, Schramm E, Richter H, et al. Shame and implicit self-concept in women with borderline personality disorder. Am J Psychiatry. 2007;164(3):500–8. 17329476 10.1176/ajp.2007.164.3.500

[31] Song X, Anderson T, Himawan L, McClintock A, Jiang Y, McCarrick S. An investigation of a cultural help-seeking model for professional psychological services with U.S. and Chinese samples. J Cross-Cult Psychol. 2019;50:1027–49.

[32] Pescosolido BA, Boyer CA. How do people come to use mental health services? Current knowledge and changing perspectives. In: A handbook for the study of mental health: Social contexts, theories, and systems. Cambridge University Press; 1999. p. 392–411.

[33] Barney LJ, Griffiths KM, Jorm AF, Christensen H. Stigma about depression and its impact on help-seeking intentions. Aust N Z J Psychiatry. 2006;40:51–4. 16403038 10.1080/j.1440-1614.2006.01741.x

[34] Schomerus G, Matschinger H, Angermeyer MC. The stigma of psychiatric treatment and help-seeking intentions for depression. Eur Arch Psychiatry Clin Neurosci. 2009;259:298–306. 19224105 10.1007/s00406-009-0870-y

[35] Schulze LN, Klinger-König J, Stolzenburg S, Wiese J, Speerforck S, Van Der Auwera-Palitschka S, et al. Shame, self-identification with having a mental illness, and willingness to seek help in northeast Germany. Psychiatry Res. 2020;285:112819. 32036156 10.1016/j.psychres.2020.112819

[36] Barta T, Kiropoulos L. The mediating role of stigma, internalized shame, and autonomous motivation in the relationship between depression, anxiety, and psychological help-seeking attitudes in multiple sclerosis. Int J Behav Med. 2023;30:133–45. 35325406 10.1007/s12529-022-10078-6 PMC9879833

[37] Gilbert P. The relationship of shame, social anxiety and depression: the role of the evaluation of social rank. Clin Psychol Psychother. 2000;7:174–89.

[38] Sznycer D, Takemura K, Delton AW, Sato K, Robertson T, Cosmides L, et al. Cross-cultural differences and similarities in proneness to shame: An adaptationist and ecological approach. Evol Psychol. 2012;10:147470491201000. 10.1177/147470491201000213 PMC3604996 22947644

[39] Bedford O, Hwang K. Guilt and shame in chinese culture: A cross-cultural framework from the perspective of morality and identity. J Theory Soc Behav. 2003;33:127–44.

[40] Clark A. Working with guilt and shame. Adv Psychiatr Treat. 2012;18:137–43.

[41] Miceli M, Castelfranchi C. Reconsidering the differences between shame and guilt. Eur J Psychol. 2018;14:710–33. 30263080 10.5964/ejop.v14i3.1564 PMC6143989

[42] Parker S, Thomas R. Psychological differences in shame vs. guilt: Implications for mental health counselors. J Ment Health Couns. 2009;31:213–24.

[43] Tangney JP, Niedenthal PM, Covert MV, Barlow DH. Are shame and guilt related to distinct self-discrepancies? A test of Higgins’s (1987) hypotheses. J Pers Soc Psychol. 1998;75:256–68. 9686463 10.1037//0022-3514.75.1.256

[44] Gilbert P, Gilbert J, Sanghera J. A focus group exploration of the impact of izzat, shame, subordination and entrapment on mental health and service use in South Asian women living in Derby. Ment Health Relig Cult. 2004;7:109–30.

[45] Wilson RW. Conformity and deviance regarding moral rules in Chinese society: A socialization perspective. In: Kleinman A, Lin T-Y, editors. Normal and Abnormal Behavior in Chinese Culture. Dordrecht: Springer Netherlands; 1981. p. 117–36.

[46] Andrews B, Qian M, Valentine JD. Predicting depressive symptoms with a new measure of shame: The Experience of Shame Scale. Br J Clin Psychol. 2002;41:29–42. 11931676 10.1348/014466502163778

[47] Zhang H, Carr ER, Garcia-Williams AG, Siegelman AE, Berke D, Niles-Carnes LV, et al. Shame and depressive symptoms: self-compassion and contingent self-worth as mediators? J Clin Psychol Med Settings. 2018;25:408–19. 29488038 10.1007/s10880-018-9548-9

[48] Hennessy EA, Johnson BT, Acabchuk RL, McCloskey K, Stewart-James J. Self-regulation mechanisms in health behavior change: a systematic meta-review of meta-analyses, 2006–2017. Health Psychol Rev. 2020;14:6–42. 31662031 10.1080/17437199.2019.1679654 PMC7571594

[49] Bagozzi RP, Verbeke W, Gavino JC. Culture moderates the self-regulation of shame and its effects on performance: The case of salespersons in the Netherlands and the Philippines. J Appl Psychol. 2003;88:219–33. 12731706 10.1037/0021-9010.88.2.219

[50] Sheikh S, Janoff-Bulman R. The, “Shoulds” and “Should Nots” of moral emotions: A self-regulatory perspective on shame and guilt. Pers Soc Psychol Bull. 2010;36:213–24. 20008966 10.1177/0146167209356788

[51] Corrigan PW, Miller FE. Shame, blame, and contamination: A review of the impact of mental illness stigma on family members. J Ment Health. 2004;13:537–48.

[52] Schibalski JV, Müller M, Ajdacic-Gross V, Vetter S, Rodgers S, Oexle N, et al. Stigma-related stress, shame and avoidant coping reactions among members of the general population with elevated symptom levels. Compr Psychiatry. 2017;74:224–30. 28236772 10.1016/j.comppsych.2017.02.001

[53] Helmert C, Fleischer T, Speerforck S, Ulke C, Altweck L, Hahm S, et al. An explorative cross-sectional analysis of mental health shame and help-seeking intentions in different lifestyles. Sci Rep. 2023;13:10825. 37402843 10.1038/s41598-023-37955-8 PMC10319876

[54] Ng E. Shame-informed Counselling and Psychotherapy. 1st ed. London: Routledge; 2020.

[55] Nagai S. Predictors of help-seeking behavior: Distinction between help-seeking intentions and help-seeking behavior. Jpn Psychol Res. 2015;57:313–22.

[56] Tomczyk S, Schomerus G, Stolzenburg S, Muehlan H, Schmidt S. Ready, willing and able? An investigation of the theory of planned behaviour in help-seeking for a community sample with current untreated depressive symptoms. Prev Sci. 2020;21:749–60. 32140825 10.1007/s11121-020-01099-2 PMC7366606

[57] Hammer JH, Spiker DA. Dimensionality, reliability, and predictive evidence of validity for three help-seeking intention instruments: ISCI, GHSQ, and MHSIS. J Couns Psychol. 2018;65:394–401. 29672088 10.1037/cou0000256

[58] Kessler RC, Barker PR, Colpe LJ, Epstein JF, Gfroerer JC, Hiripi E, et al. Screening for serious mental illness in the general population. Arch Gen Psychiatry. 2003;60:184. 12578436 10.1001/archpsyc.60.2.184

[59] Kang Y, Guo W, Xu H, Chen Y, Li X, Tan Z, et al. The 6-item Kessler psychological distress scale to survey serious mental illness among Chinese undergraduates: Psychometric properties and prevalence estimate. Compr Psychiatry. 2015;63:105–12. 26555498 10.1016/j.comppsych.2015.08.011

[60] Harder DW, Rockart L, Cutler L. Additional validity evidence for the harder personal feelings questionnaire-2 (PFQ2): A measure of shame and guilt proneness. J Clin Psychol. 1993;49:345–8. 8315036 10.1002/1097-4679(199305)49:3<345::aid-jclp2270490307>3.0.co;2-y

[61] Harder DH, Zalma A. Two promising shame and guilt scales: A construct validity comparison. J Pers Assess. 1990;55:729–45. 2280336 10.1080/00223891.1990.9674108

[62] Erkut S. Developing multiple language versions of instruments for intercultural research. Child Dev Perspect. 2010;4:19–24. 21423824 10.1111/j.1750-8606.2009.00111.x PMC3060794

[63] Lubben J, Blozik E, Gillmann G, Iliffe S, Von Renteln KW, Beck JC, et al. Performance of an abbreviated version of the lubben social network scale among three European community-dwelling older adult populations. Gerontologist. 2006;46:503–13. 16921004 10.1093/geront/46.4.503

[64] Hamilton HA, Paglia-Boak A, Wekerle C, Danielson AM, Mann RE. Psychological distress, service utilization, and prescribed medications among youth with and without histories of involvement with child protective services. Int J Ment Health Addict. 2011;9:398–409.

[65] Prochaska JJ, Sung H, Max W, Shi Y, Ong M. Validity study of the K6 scale as a measure of moderate mental distress based on mental health treatment need and utilization. Int J Methods Psychiatr Res. 2012;21:88–97. 22351472 10.1002/mpr.1349 PMC3370145

[66] Hong Y, Li X, Stanton B, Lin D, Fang X, Rong M. Too costly to be ill: health care access and health seeking behaviors among rural-to-urban migrants in China. World Health Popul. 2006;8(2):22. 18277099 10.12927/whp.2006.18280 PMC2249561

[67] Gondek D, Kirkbride JB. Predictors of mental health help-seeking among Polish people living in the United Kingdom. BMC Health Serv Res. 2018;18:693. 30189870 10.1186/s12913-018-3504-0 PMC6127920

[68] Millán-Franco M, Gómez-Jacinto L, Hombrados-Mendieta I, González-Castro F, García-Cid A. The effect of length of residence and geographical origin on the social inclusion of immigrants. Psychosoc Interv. 2019;28:119–30.

[69] De Boer AGEM, Wijker W, De Haes HCJM. Predictors of health care utilization in the chronically ill: a review of the literature. Health Policy. 1997;42:101–15. 10175619 10.1016/s0168-8510(97)00062-6

[70] Lam LCW, Wong CSM, Wang MJ, Chan WC, Chen EYH, Ng RMK, et al. Prevalence, psychosocial correlates and service utilization of depressive and anxiety disorders in Hong Kong: the Hong Kong Mental Morbidity Survey (HKMMS). Soc Psychiatr Psychiatr Epidemiol. 2015;50:1379–88. 10.1007/s00127-015-1014-5 25660760

[71] Jiang Y, Zhang J, Jin X, Ando R, Chen L, Shen Z, et al. Rural migrant workers’ intentions to permanently reside in cities and future energy consumption preference in the changing context of urban China. Transp Res Part Transp Environ. 2017;52:600–18.

[72] Quach AS, Hall DH. Chinese American Attitudes toward Therapy: Effects of Gender, Shame, and Acculturation. Int J Humanit Soc Sci. 2013;3(12):209–22.

[73] Seah R, Dwyer K, Berle D. Was it me? The role of attributions and shame in posttraumatic stress disorder (PTSD): A systematic review. Trends Psychol. 2023. 10.1007/s43076-023-00315-6.

[74] Markova V, Sandal GM, Pallesen S. Immigration, acculturation, and preferred help-seeking sources for depression: comparison of five ethnic groups. BMC Health Serv Res. 2020;20:648. 32652988 10.1186/s12913-020-05478-x PMC7353801

[75] Vogel DL, Wester SR, Larson LM. Avoidance of counseling: Psychological factors that inhibit seeking help. J Couns Dev. 2007;85:410–22.

[76] Lickel B, Kushlev K, Savalei V, Matta S, Schmader T. Shame and the motivation to change the self. Emotion. 2014;14:1049–61. 25401288 10.1037/a0038235

[77] De Hooge IE, Zeelenberg M, Breugelmans SM. A functionalist account of shame-induced behaviour. Cogn Emot. 2011;25:939–46. 21824031 10.1080/02699931.2010.516909

[78] Efrati Y. Adolescents with a disposition toward compulsive sexual behavior: The role of shame in willingness to seek help and treatment. Sex Addict Compulsivity. 2018;25:28–45.

[79] Jacquet J, Hauert C, Traulsen A, Milinski M. Shame and honour drive cooperation. Biol Lett. 2011;7:899–901. 21632623 10.1098/rsbl.2011.0367 PMC3210662

[80] Ran M-S, Hall BJ, Su TT, Prawira B, Breth-Petersen M, Li X-H, et al. Stigma of mental illness and cultural factors in Pacific Rim region: a systematic review. BMC Psychiatry. 2021;21:8. 33413195 10.1186/s12888-020-02991-5 PMC7789475

[81] Lawthom R, Kagan C, Baines S, Lo S, Sham S, Mok L, et al. Experiences of forced labour amongst Chinese migrant workers: exploring the context of vulnerability and protection. Int J Work Organ Emot. 2013;5:261.

[82] Chen J. Some people may need it, but not me, not now: seeking professional help for mental health problems in urban China. Transcult Psychiatry. 2018;55:754–74. 30113276 10.1177/1363461518792741

[83] Gong AT, Furnham A. Mental health literacy: Public knowledge and beliefs about mental disorders in mainland China: Mental health literacy in China. PsyCh J. 2014;3:144–58. 26271766 10.1002/pchj.55

[84] Tangney JP, Miller RS, Flicker L, Barlow DH. Are shame, guilt, and embarrassment distinct emotions? J Pers Soc Psychol. 1996;70:1256–69. 8667166 10.1037//0022-3514.70.6.1256

[85] Gaebel W, Kowitz S, Zielasek J. The DGPPN research project on mental healthcare utilization in Germany: inpatient and outpatient treatment of persons with depression by different disciplines. Eur Arch Psychiatry Clin Neurosci. 2012;262:51–5. 10.1007/s00406-012-0363-2 22940745

[86] Huang W, Long H, Li J, Tao S, Zheng P, Tang S, et al. Delivery of public health services by community health workers (CHWs) in primary health care settings in China: a systematic review (1996–2016). Glob Health Res Policy. 2018;3:18. 29992191 10.1186/s41256-018-0072-0 PMC5989355

[87] Rickwood D, Deane FP, Wilson CJ, Ciarrochi J. Young people’s help-seeking for mental health problems. Aust E-J Adv Ment Health. 2005;4:218–51.

