# Supplementary material for: Mental Health Help-Seeking Among Young Internal Migrants in China: Shame as a Double-Edged Sword
Source: Psychiatr Q. 2025 Apr 15;96(4):829–45. doi: 10.1007/s11126-025-10127-9 (PMC12647222; doi:10.1007/s11126-025-10127-9)
Supplement: Supplementary file 1 — Supplementary file1 (DOCX 27 KB) [file 11126_2025_10127_MOESM1_ESM.docx]

Supplementary Table 1. Regression models for professional help-seeking intention, excluding participants who have sought help before

|  | b | SE | *p* |
| --- | --- | --- | --- |
| Psychological stress | -0.75 | 0.16 | 0.000 |
| Shame | -1.15 | 0.20 | 0.000 |
| Psychological stress × Shame | 1.44 | 0.30 | 0.000 |
| *Predisposing factors* |  |  |  |
| Age | 0.03 | 0.06 | 0.610 |
| Gender | 0.19 | 0.10 | 0.064 |
| Married | 0.18 | 0.12 | 0.136 |
| Vocational education | 0.13 | 0.14 | 0.359 |
| Tertiary education | 0.01 | 0.15 | 0.926 |
| Hukou status | -0.13 | 0.14 | 0.343 |
| Length of living in the current city | 0.06 | 0.06 | 0.319 |
| *Enabling factors* |  |  |  |
| Income | -0.07 | 0.06 | 0.225 |
| Insurance coverage | 0.15 | 0.24 | 0.529 |
| Social network | 0.27 | 0.07 | 0.000 |
| *Need factor* |  |  |  |
| General health condition | -0.05 | 0.06 | 0.382 |

Supplementary Figure 1. The moderating effect of shame in the relationship between psychological stress and professional help-seeking intention, excluding participants who have sought help before
